# Duloxetine compared with fluoxetine and venlafaxine: use of meta-regression analysis for indirect comparisons

**DOI:** 10.1186/1471-244X-6-30

**Published:** 2006-07-24

**Authors:** Laurent Eckert, Christophe Lançon

**Affiliations:** 1 Unit 669, Institut Nationale de la Santé et de la Recherche Médicale (INSERM), F-75014, Paris, France; and the Univeristy of Paris-Sud 11, F-94000, Le Kremlin Bicêtre, France; 2 Hopital Sainte-Marguerite, 270, boulevard Sainte-Marguerite, 13274 Marseille, France

## Abstract

**Background:**

Data comparing duloxetine with existing antidepressant treatments is limited. A comparison of duloxetine with fluoxetine has been performed but no comparison with venlafaxine, the other antidepressant in the same therapeutic class with a significant market share, has been undertaken. In the absence of relevant data to assess the place that duloxetine should occupy in the therapeutic arsenal, indirect comparisons are the most rigorous way to go.

We conducted a systematic review of the efficacy of duloxetine, fluoxetine and venlafaxine versus placebo in the treatment of Major Depressive Disorder (MDD), and performed indirect comparisons through meta-regressions.

**Methods:**

The bibliography of the Agency for Health Care Policy and Research and the CENTRAL, Medline, and Embase databases were interrogated using advanced search strategies based on a combination of text and index terms. The search focused on randomized placebo-controlled clinical trials involving adult patients treated for acute phase Major Depressive Disorder. All outcomes were derived to take account for varying placebo responses throughout studies. Primary outcome was treatment efficacy as measured by Hedge's *g *effect size. Secondary outcomes were response and dropout rates as measured by log odds ratios. Meta-regressions were run to indirectly compare the drugs. Sensitivity analysis, assessing the influence of individual studies over the results, and the influence of patients' characteristics were run.

**Results:**

22 studies involving fluoxetine, 9 involving duloxetine and 8 involving venlafaxine were selected. Using indirect comparison methodology, estimated effect sizes for efficacy compared with duloxetine were 0.11 [-0.14;0.36] for fluoxetine and 0.22 [0.06;0.38] for venlafaxine. Response log odds ratios were -0.21 [-0.44;0.03], 0.70 [0.26;1.14]. Dropout log odds ratios were -0.02 [-0.33;0.29], 0.21 [-0.13;0.55]. Sensitivity analyses showed that results were consistent.

**Conclusion:**

Fluoxetine was not statistically different in either tolerability or efficacy when compared with duloxetine. Venlafaxine was significantly superior to duloxetine in all analyses except dropout rate. In the absence of relevant data from head-to-head comparison trials, results suggest that venlafaxine is superior compared with duloxetine and that duloxetine does not differentiate from fluoxetine.

## Background

Duloxetine is a selective serotonin and norepinephrine reuptake inhibitor (SNRI) that claims greater affinity for the serotonin and norepinephrine transporters compared with venlafaxine [1,2]. The efficacy and safety of duloxetine in the treatment of major depressive disorder (MDD) in adults (18–65 years) has been evaluated in 9 phase II and III clinical trials [3-5]. All were randomized, double blind, placebo-controlled studies with doses ranging from 40 to 120 mg/day in the acute treatment of MDD. Results have shown that duloxetine provided relief from psychological symptoms of depression compared with placebo. Six of the above studies used an active comparator: either fluoxetine or paroxetine. None, however, was designed and powered for direct head-to-head comparison between duloxetine and the active comparator. Inclusion of a selective serotonin reuptake inhibitor (SSRI) was intended only to show non-inferiority of duloxetine. No trial has used venlafaxine, the other marketed SNRI, as an active comparator.

The amount of data comparing duloxetine with existing antidepressant treatments is quite limited. The lack of direct comparisons between the recommended daily dose (60 mg) and an active comparator was criticised in a recent evaluation of duloxetine by the Committee for Medicinal Products for Human Use (CHMP) [6]. Assessments of the benefit/risk ratio of a new drug compared with a standard drug at an adequate dose are generally required and it is recommended that clinical trials be conducted not only against placebo, but also against active comparators [7]. The aim of such studies may be to show superiority over the active comparator or to demonstrate that at least a similar balance between benefit and risk exists when the drug of interest is compared with another acknowledged standard antidepressant.

In the absence of head-to-head randomized studies, indirect comparisons can be made between molecules. Clinical trials frequently compare efficacy of a drug versus placebo in the treatment of MDD. Less frequent, however, are head-to-head comparisons. Indirect comparisons taking into account all available placebo-controlled studies are capable of obtaining an effect size and a confidence interval of the difference between two compounds. The algorithm used gives results adjusted for discrepancies in sociodemographics, settings and designs.

After conducting a systematic review of the efficacy of duloxetine, fluoxetine and venlafaxine versus placebo in the treatment of MDD we performed an indirect comparison of the benefits of duloxetine versus fluoxetine and venlafaxine. We used meta-regression analysis to test whether or not differences in effectiveness (which cannot be explained by the differences in settings only) exist between fluoxetine and duloxetine on one hand and venlafaxine and duloxetine on the other.

## Methods

### The analyses sets

We used advanced search strategies based on a combination of text and index terms to interrogate the CENTRAL, Medline and Embase databases as well as the bibliography of the US Agency for Health Care Policy and Research (AHCPR). The bibliography from the AHCPR is an exhaustive literature search (both published and non-published) of trials in depression up to 1999.

Selection criteria were: study reporting HAMD results in randomised trials with a placebo arm, involving adult patients suffering from MDD (as assessed by DSM (III, III-R, IV)) treated in acute phase with either fluoxetine, venlafaxine, duloxetine. Excusion criteria were presence of comorbidities; absence of the HAMD scale; involving adolescents, children or elderly; absence of randomisation and absence of a placebo arm.

These criteria were considered sufficient to retrieve all studies of interest to be included in the analysis set.

Two research assistants independently selected papers by reading the abstract and, if necessary, the entire article to assess eligibility and data extraction. Careful re-reading of the papers resolved differences between each author analysis set and letters were sent to corresponding authors in the attempt to reduce missing data.

Publication bias was assessed drawing funnel plots, and Egger Test was used to test funnel plot asymmetry.

### Statistical outcomes

Because different trials do not necessarily use the same scale and/or version for assessing efficacy, an effect size was derived from the primary outcome of each study (either HAM-D 17 21 or 24). This enabled deriving a common effect measure across studies that used different scales. The effect size was Hedge's *g *(a Standardised Response Mean estimator), which was corrected for small sample size bias. To compute an effect size, both the mean and an estimate of dispersion (variance, standard deviation) have to be present. When the dispersion was missing, data was imputed using the sample size weighted method [8]. If both mean and dispersion were missing, the study was removed from the analysis set.

The computed effect sizes were adjusted for severity at baseline to account for differences in patients' groups (selection bias).

The effect size was defined as the difference between the mean change in depression scale score from baseline to end-of-study in the active arm and the mean change in depression scale score from baseline to end of study in the placebo arm; divided by the standard deviation of the difference.

Other endpoints were response and dropout rates. Response was defined as a reduction of at least 50% in the HAM-D score from baseline. Dropouts were considered regardless of cause, which gave a rough indicator of the tolerability and safety and efficacy of the treatment. In other words, dropouts were an indicator of failures of the present therapy.

The response and dropouts rates were analysed using log-odds ratios. A log-odds ratio equal to zero indicated that there was no statistical difference between the two compared groups. Considering the response rate, a value greater than zero indicated that more patients in the treatment group were classified as responders, and therefore that the treatment was better compared with the reference (placebo or duloxetine). A value lower than zero indicated that the reference (placebo or duloxetine) was better. Regarding dropouts, a value greater than zero indicated that more patients in the reference group (placebo or duloxetine) withdrew, and therefore that the treatment was better (in terms of efficacy and/or safety) compared with the reference (placebo or duloxetine). A value lower than zero indicated that treatment was less effective or less tolerated than the reference (placebo or duloxetine).

### Statistical methods

Random-effect meta-analyses were computed for each outcome and each treatment compared with placebo. Mean age, mean percentage of male, mean study duration and range of dosage were computed for each treatment.

Following recommendations by Glenny *et al*. [9] and van Houwelingen *et al*. [10], a mixed procedure was run. This enabled handling studies with more than two arms (typically when different dosages are included in the same study), as well as studies presenting two drugs in the same trial (two trials assessed the effectiveness of duloxetine versus placebo and were fluoxetine controlled). The method used is a weighted least squares algorithm which iteratively computes a between-study variance while keeping each within-study variance constant. Therefore, what are modelled by default (when no adjustment is made) are drug effect (an antidepressant effect of the drugs) and drug-specific effect. The drug specific effect is the effect tested between the two treatments compared.

The models were computed under SAS PROC MIXED [11]. This procedure gives also good coverage for confidence intervals according to van Houwelingen *et al*. [10]. As in van Houwelingen *et al*., [10] Wald confidence intervals were used.

Sensitivity analyses were planned a priori and included: Performing several adjustments. The variables chosen *a priori *as having a potential influence over the outcome of a study were age, male percentage, duration of study and dosage. Robustness was then assessed observing the variation in the estimation of the outcome, its corresponding confidence interval, as well as the size of the estimated residual between-study variance  [10].An adjustment over the fact that the effect size was imputed was also run (in case the dispersion had to be imputed to compute an effect size). To assess its influence over the results, studies were removed from the analysis set one at a time. A *post hoc*. sensitivity analysis was run on a subgroup of fluoxetine studies excluding the studies where the number of patients was below 20.

The following rules were applicable for all computed models:

• In case an adjustment factor was missing, it was imputed by the corresponding weighted mean computed with available data.

• Influence of missing data was computed through sensitivity analyses by removing the studies where the data was missing.

• In the event that an outcome was missing and no reply was received from the letters sent, the study was removed from the analysis set for the particular analysis for which the outcome was missing.

## Results

No precise answers were received from the letters sent to corresponding authors; therefore, the number of missing data remained unchanged.

### Individual studies results

**Figure 1:**
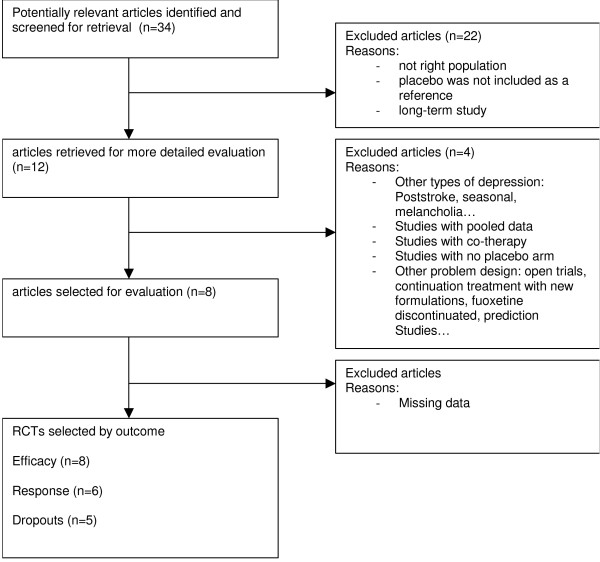
Diagram Flow for duloxetine.

*For duloxetine*, 8 publications showing results for 9 trials (each with varying characteristics) were selected, [Figure 1]. [Table 1] matches the publications with the information available from each trial. Mean age varied from 41 to 45 and the percentage of males varied from 25 to 40%. Duration of treatment varied from 8 to 9 weeks and dosages (fixed or variable) were from 40 to 120 mg per day. The effect size comparing duloxetine to placebo was -0.29(0.15). The response and dropouts log odds ratio were 0.58(0.18) and -0.02(0.32) respectively. The funnel plot shape cannot rule out the possibility of a publication bias; see [Figure 4]. The Funnel plot was not statistically significantly asymmetrical according to the Egger test (p = 0.9).

**Table 1:** Selected studies presentation for duloxetine

Study	Inclusion criteria	Age	Percent male	Duration (weeks)	Treatment	Dosage (mg/day)	Patients per arm	Effect size (SD)	Response (%)	Dropouts (%)
Goldstein et al. [9]	DSM-IV, Age 18–65 HAM-D_17 _≥ 15, CGI-S ≥ 4	42	0.34	8	Duloxetine Placebo	40–120	70 70	-0.3(0.03)	0.64 0.48	0.34 0.34
Detke et al. [10]	DSM-IV, Age ≥ 18 HAM-D_17 _≥ 15, CGI-S ≥ 4	42	0.33	9	Duloxetine Placebo	60	123 122	-0.6(0.02)	0.62 0.29	
Detke et al. [12]	DSM-IV, Age ≥ 18 HAM-D_17 _≥ 15, CGI-S ≥ 4	41	0.31	9	Duloxetine Placebo	60	128 139	-0.2(0.02)	0.65 0.42	0.39 0.35
Detke et al. [13]	DSM-IV, Age ≥ 18 HAM-D_17 _≥ 15, CGI-S ≥ 4	45 43	0.26 0.25	8	Duloxetine Duloxetine Placebo	80 120	95 93 93	-0.3(0.02) -0.6(0.02)	0.65 0.71 0.44	0.13 0.10 0.19
Goldstein et al. [14]	DSM-IV, Age ≥ 18 HAM-D_17 _≥ 15, CGI-S ≥ 4	41 41	0.4 0.38	8	Duloxetine Duloxetine Placebo	40 80	86 91 89	-0.4(0.02) -0.5(0.02)	0.54 0.60 0.30	0.36 0.42 0.42
Greist et al. [4] Falissard et al. [3]	DSM-IV, Age ≥ 18			8	Duloxetine Placebo	120	82 75	-0.1(0.03)		
Greist et al. [4] Falissard et al. [3]	DSM-IV, Age ≥ 18			8	Duloxetine Duloxetine Placebo	40 80	91 84 90	-0.2(0.02) -0.4(0.02)		
Greist et al. [4] Falissard et al. [3]	DSM-IV, Age ≥ 18			8	Duloxetine Duloxetine Placebo	80 120	93 103 99	-0.3(0.02) -0.3(0.02)		
Brannan et al. [5]	DSM-IV, Age ≥ 18 HAM-D_17 _≥ 15, CGI-S ≥ 4	41	0.35	9	Duloxetine Placebo	60	141 141	-0.2(0.02)	0.42 0.40	0.16 0.09
Globally		42 (1)	0.33	8 (0.4)	Duloxetine Placebo	20–120	1280 918	-0.29(0.05)	0.58(0.09) **	-0.02(0.16) **

** Log Odds ratios (SD)

**Figure 2:**
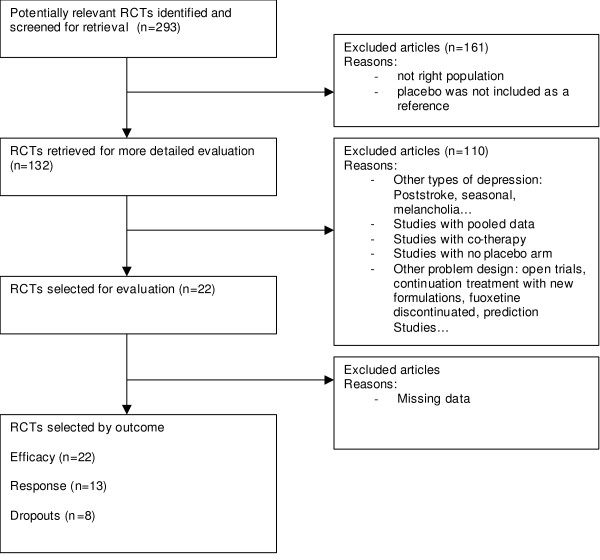
Diagram Flow for fluoxetine.

*For fluoxetine*, 22 papers were selected [Figure 2], presenting a rather heterogeneous picture [Table 2]. Mean age varied from 33 to 47 and the percentage of males varied from 26 to 57%. Duration of treatment varied from 5 to 12 weeks and dosages (fixed or variable) were from 20 to 80 mg per day. It is worth noting that some studies include few patients (from 5 to 169). The effect size comparing fluoxetine to placebo was -0.46(0.52). The response and dropouts log odds ratio were 0.37(0.32) and -0.02(0.23), respectively. A positive point worth noting is that publication bias is shown to be minimised (see Figure 4). This figure shows the typical conic shape centred over the value estimated which indicates little or no bias. The Funnel plot was not statistically significantly asymmetrical according to the Egger test (p = 0.4).

**Table 2:** Selected studies presentation for fluoxetine

Study	Inclusion criteria	Age	Percent male	Duration (weeks)	Treatment	Dosage (mg/day)	Patients per arm	Effect size (SD)	Response (%)	Dropout (%)
Fabre et al. [15]	Age 21–70 HAM-D ≥ 20			5	Fluoxetine Placebo	40–80	22 26	-0.8 (0.1)		0.50 0.44
Stark et al. [16]	DSM-III, Age 18–70 HAM-D_21 _≥ 20	40	0.32	6	Fluoxetine Placebo	20–80	185 169	-0.3(0.01)	0.63 0.38	0.37 0.45
Cohn et al. [17]	DSM-III, Age 20–64 HAM-D ≥ 20	41	0.40	6	Fluoxetine Placebo	20–80	54 57	-1.3(0.04)	0.72 0.30	0.30 0.72
Fieve et al. [18]	DSM-III, Age 18–65 HAM-D_21 _≥ 20		0.57	6	Fluoxetine Fluoxetine Fluoxetine Placebo	5 20 40	14 13 13 9	-0.5 (0.1)		
Rickels et al. [19]	DSM-III, Age 21–70 HAM-D ≥ 20	47.2	0.21	5	Fluoxetine Placebo	20–80	18 24	-0.7 (0.2)	0.90 0.39	
Goodnick et al. [20]	DSM-III, Age 18–65 HAM-D ≥ 116		0.54	7	Fluoxetine Placebo	20–60	30 5	-1.1(0.3) 0.1(0.2)		
Wernicke et al. [21]	DSM-III, Age 18–65 HAM-D ≥ 20	39.8	0.43	6	Fluoxetine Fluoxetine Fluoxetine Placebo	20 40 60	97 97 103 48	-0.5(0.03) -0.5(0.03) -0.2(0.03)	0.53 0.61 0.48 0.27	0.38 0.40 0.55 0.44
Fabre et al. [22]	DSM-III, Age 18–65 HAM-D ≥ 14			6	Fluoxetine Fluoxetine Fluoxetine Placebo	20 40 60	22 25 25 12	-2.1 (0.4) -1.5 (0.3) -2.3 (0.5)	0.67 0.31 1 0	
Wernicke et al. [23]	DSM-III, Age 18–65 HAM-D ≥ 14	39	0.37	6	Fluoxetine Fluoxetine Fluoxetine Placebo	5 20 40	94 91 92 77	-0.5(0.02) -0.4(0.02) -0.5(0.02)	0.54 0.64 0.65 0.33	
Harto et al. [24]	DSM-III, Age 18–65 HAM-D ≥ 20	38.4 43.8 36.4	0.37 0.37 0.37	6	Fluoxetine Fluoxetine Fluoxetine Placebo	5 20 40	8 10 9 8	1 (0.3) 1.1 (0.3) 0.9 (0.3)		
Byerley et al. [25]	DSM-III HAM-D_21 _≥ 20	38.9	0.31	6	Fluoxetine Placebo	40–80	32 29	-1.1 (0.1)		0.38 0.45
Muijen et al. [26]	Research Diagnostic Criteria, Age 18–65 HAMD-D17 ≥ 17	35.8	0.37	6	Mianserin Fluoxetine Placebo	20 20	27 26 28	-1.2 (0.2)	0.55 0.23	0.46 0.43
Feighner et al. [27]	DSM-III, Age 18–70 HAMD-D21 ≥ 20, RSDM ≥ 8 and RSDM ≥ Covi	45	0.26	6	Imipramine Fluoxetine Placebo	150 80 (median)	46 51 48	-0.3(0.04)		0.51 0.68
Dunlop et al. [28]	DSM-III, Age 18–65 HAMD-D ≥ 14 and RSDM >Covi	39.3 39.3 39.3		6	Fluoxetine Fluoxetine Fluoxetine Placebo	20 40 60	103 99 97 56	-0.1(0.03) -0.03 (0.03) 0.1(0.03)	0.53 0.51 0.59 0.36	0.35 0.41 0.39 0.34
Valducci et al. [29]	DSM-III-R, Age 19–67 HAMD-D >18		0.43	8	Fluoxetine Placebo	20	20 20	-0.9 (0.1)	0.70 0.25	
Heiligenstein et al. [31]	DSM-III-R, Age 18–65 HAMD-D17 ≥ 15	44.4		8	Fluoxetine Placebo	20	46 43	0.1 (0.1) -0.9 (0.1)	0.61 0.41	
Sramek et al. [32]	DSM-III-R, Age 18–65 HAMD-D24 ≥ 21 with item 1 ≥ 2, HAM-A ≤ 18, HAMD-D24 <HAM-A, RSDM ≥ 8 and RSDM >Covi	33.9	0.40	9	ABT-200 Fluoxetine Placebo	160–320 20	72 72 72	-0.3(0.03)		0.17 0.13
Fava et al. [33]	DSM-III-R, Age (mean 41,3) HAMD-D17 ≥ 18, Raskin depression score ≥ 8 and superior to Raskin anxiety score	41.3	0.49	12	Paroxetine Fluoxetine Placebo	20–50 20–80	55 54 19	0.1 (0.1)	0.57 0.53	
Rudolph et al. [34]	DSM-IV, Age > 18 HAMD-D21 ≥ 20	40	0.33	8	Venlafaxine XR Fluoxetine Placebo	75–225 20–60	100 103 98	-0.2(0.02)	0.50 0.42	0.27 0.21
Coleman et al. [35]	DSM-IV, Age 18–76 HAMD-D21 ≥ 20	37.1		8	Bupropion SR Fluoxetine Placebo	150–400 20–60	150 154 152	-0.2(0.01)	0.57 0.50	0.37 0.33
Goldstein et al. [9]	DSM-IV, Age 18–65 HAMD-D17 ≥ 15 and CGI-S ≥ 4	39.7	0.35	8	Duloxetine Fluoxetine Placebo	40–120 20	70 33 70	-0.2(0.04)	0.45 0.39	0.36 0.34
Silverstone et al. [42]	DSM-IV, Age ≥ 18 HAMD-D21 ≥ 20 (on the first 17 items), Covi ≥ 8	43.2	0.4	12	Fluoxetine Venlafaxine XR Placebo	20–60 75–225	121 128 119	-0.6(0.02)	0.62 0.43	0.26 0.40
Globally		40 ± 3	0.39	7 ± 2	Fluoxetine Placebo	20–80	2078 1093	-0.46(0.11)	0.37(0.09) **	-0.02(0.09) **

** Log Odds ratios (SD)

**Figure 3:**
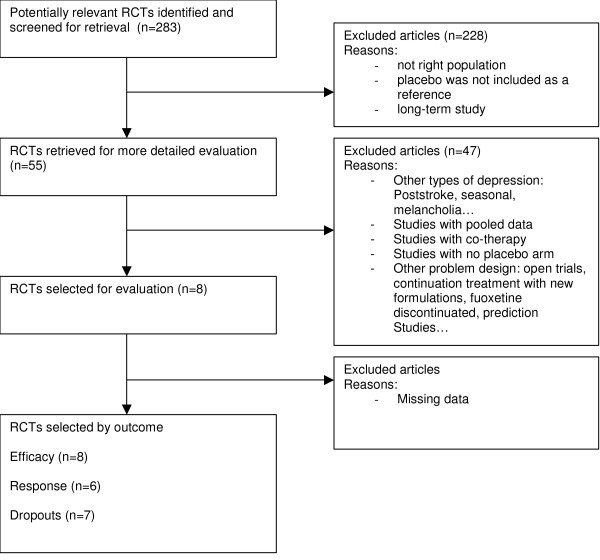
Diagram Flow for venlafaxine.

**Figure 4:**
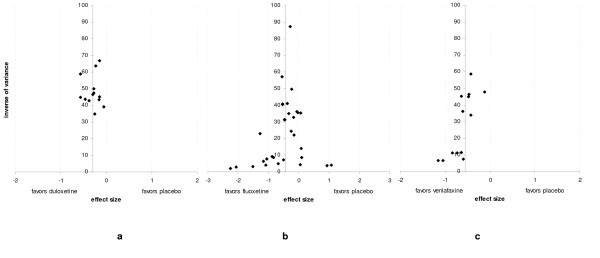
**Funnel Plots**. a duloxetine b fluoxetine c venlafaxine

*For venlafaxine*, 8 papers were selected, see [Figure 3], with the following characteristics [Table 3]. Mean age varied from 40 to 46 and the percentage of males varied from 31 to 60%. Duration of treatment varied from 6 to 12 weeks and the dosages (fixed or variable) were from 75 to 225 mg per day. The effect size comparing venlafaxine to placebo was -0.51(0.20). The response and dropouts log odds ratio were 1.28(0.64) and -0.25(0.32), respectively. The funnel plot shape cannot rule out the possibility of all publication bias [Figure 4]. The Funnel plot was not statistically significantly asymmetrical according to the Egger test (p = 0.1).

**Table 3:** Selected studies presentation for venlafaxine

Study	Inclusion criteria	Age	Percent male	duration (weeks)	Treatment	Dosage (mg/day)	Patients per arm	Effect size (SD)	Response (%)	Dropouts (%)
Khan et al. [36]	DSM-III, HAMD-D ≥ 20	41 41 41	0.44 0.44 0.44	6	Venlafaxine Venlafaxine Venlafaxine Placebo	75 75–225 150–375	23 22 22 26	-0.8(0.1) -0.6(0.1) -0.7(0.1)		0.21 0.15
Schweizer et al. [37]	DSM-III-R, Age 24–63 HAMD-D ≥ 20	46 46 46	0.60 0.60 0.60	6	Venlafaxine Venlafaxine Venlafaxine Placebo	75 225 375	15 15 14 16	-0.6(0.1) -1.2(0.2) -1.1(0.2)		0.43 0.57
Cunningham et al. [38]	DSM-III-R, Age ≥ 18 HAMD-D ≥ 20	41	0.33	6	Venlafaxine Trazodone Placebo	25–200 50–400	72 77 76	-0.4(0.03)	0.72 0.55	0.29 0.36
Schweizer et al. [39]	DSM-III-R, Age≥ 18 HAMD-D21 ≥ 20	41	0.31	6	Imipramine Venlafaxine Placebo	75–225 75–225	73 73 78	-0.6(0.03)	0.77 0.47	0.36 0.27
Cunningham et al. [40]	DSM-III-R, Age ≥ 18 HAMD-D ≥ 20	40 43	0.38 0.39	12	Venlafaxine XR Venlafaxine IR Placebo	75–150 75–150	97 96 100	-0.5(0.02) -0.5(0.02)	0.68 0.52 0.31	0.29 0.40 0.41
Thase et al. [41]	DSM-IV, Age ≥ 18 HAMD-D21 ≥ 20	40	0.39	8	Venlafaxine XR Placebo	75–225	91 100	-0.6(0.02)	0.58 0.29	0.27 0.40
Rudolph et al. [34]	DSM-IV, Age ≥ 18 HAMD-D21 ≥ 20	40	0.32	8	Fluoxetine Venlafaxine XR Placebo	20–60 75–225	103 100 98	-0.1(0.02)	0.57 0.42	0.19 0.21
Silverstone et al. [42]	DSM-IV, Age ≥ 18 HAMD-D21 ≥ 20 (on the first 17 items), Covi ≥ 8	41	0.39	12	Fluoxetine Venlafaxine XR Placebo	20–60 75–225	121 128 119	-0.4(0.02)	0.67 0.43	0.29 0.40
Globally	NA	42 ± 2	0.43	8 ± 3	Venlafaxine Placebo	75–225	768 613	-0.51(0.07)	1.28(0.26) **	-0.25(0.12) **

** Log Odds ratios (SD)

### Meta-regressions: duloxetine compared with active comparators

**Figure 5:**
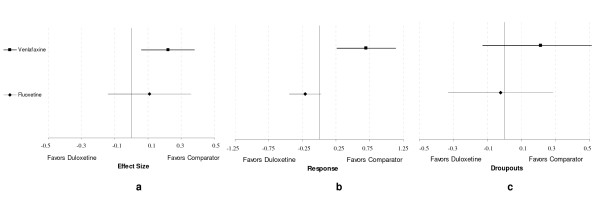
**Results: duloxetine compared with fluoxetine and venlafaxine**. a efficacy (effect size scale) b response (log odds ratio) c dropouts (log odds ratio)

*For duloxetine compared with fluoxetine*, the estimated effect size was 0.11 [-0.14;0.36] for the treatment effect (Figure 5a). The estimated response log odds ratio was -0.21 [-0.44;0.03] (Figure 5b) (only 13 fluoxetine studies and 6 duloxetine studies were included because of missing data) with a corresponding odds ratio of 0.81. The estimated dropouts log odds ratio was -0.02 [-0.33;0.29] (Figure 5c) (only 8 fluoxetine studies and 5 duloxetine studies were included because of missing data). None of these results *vs*. fluoxetine were significant, although a trend can be seen in favor of duloxetine in term of number of responders.

*For duloxetine compared with venlafaxine*, the estimated effect size was 0.22 [0.06;0.38] for the treatment effect (Figure 5a), demonstrating a significant better efficacy of venlafaxine compared with duloxetine. The estimated response log odds ratio was 0.70 [0.26;1.14] also significantly different in favour of venlafaxine (Figure 5b) (only 6 venlafaxine studies and 6 duloxetine studies were included because of missing data). The estimated dropout log odds ratio was 0.21 [-0.13;0.55] (Figure 5c) (only 7 venlafaxine and 5 duloxetine studies were included because of missing data). Venlafaxine seem more efficacious both in reduction of symptoms and in term of number of responders (the corresponding odds ratio is 2.01) for a similar safety profile.

### Sensitivity analyses

*For duloxetine compared with fluoxetine*, *cf*. [Table 4] either investigating the primary outcome (efficacy as measured by derived HAMD scale) or the response factor, the results were stable through adjustments, no amelioration in the adjustment was reached (the residual between-study variance estimate remained approximately constant), and confidence intervals remained large and stable. The effect size of the best prediction (smallest residual between-study variance) was 0.12 [-0.14;0.38]. The odds ratio of the response factor varied from 0.81 to 0.95, favouring numerically duloxetine in every analysis and reaching borderline significance when the estimate was close to 0.81. The residual between-study variance was constant. Concerning the dropout factor, the odds ratio varied from 1.21 to 1.40, numerically favouring fluoxetine in every analysis. Adjusting for duration of the study revealed a significant advantage in favour of fluoxetine (corresponding odds ratio 1.40). This advantage is borderline significant when adjusting for duration (corresponding odds ratio 1.36). The residual between-study variance was constant.

**Table 4:** Sensitivity analyses: adjustment for confounding factors

	EFFECT				RESPONSE				DROPOUTS			
	
	mean	Confidence interval		Est. bet. st. variance*	mean	Confidence interval		Est. bet. st. variance*	value	Confidence interval		Est. bet. st. variance*
AGE												
venlafaxine	0.25	0.11	0.40	0.002	0.90	0.49	1.31	0.049	0.26	-0.09	0.61	0.000
Fluoxetine	0.13	-0.14	0.39	0.097	-0.10	-0.37	0.17	0.000	0.23	-0.14	0.60	0.000
MALE PERCENT												
venlafaxine	0.16	-0.01	0.33	0.008	0.66	0.21	1.12	0.096	0.23	-0.13	0.59	0.001
Fluoxetine	0.12	-0.14	0.38	0.010	-0.17	-0.43	0.08	0.000	0.31	-0.02	0.64	0.000
DURATION												
venlafaxine	0.23	0.07	0.39	0.009	0.56	0.18	0.95	0.047	0.13	-0.23	0.48	0.000
Fluoxetine	0.10	-0.16	0.36	0.100	-0.21	-0.45	0.03	0.000	0.34	0.02	0.67	0.000
DOSAGE												
venlafaxine	0.22	0.06	0.37	0.008	0.70	0.26	1.15	0.096	0.22	-0.12	0.56	0.000
Fluoxetine	0.19	-0.10	0.48	0.094	-0.05	-0.38	0.27	0.000	0.19	-0.19	0.57	0.000
IMPUTATION												
venlafaxine	0.22	0.06	0.37	0.008	0.74	0.29	1.18	0.093	0.18	-0.18	0.54	0.000
Fluoxetine	0.11	-0.14	0.36	0.096	-0.20	-0.44	0.04	0.000	0.27	-0.06	0.60	0.000

* estimated between study variance

Whatever the parameter of interest or the adjustment factor considered, the fact that variances were imputed did not change the conclusions.

When removing studies one at a time in the analysis set, the conclusions didn't change except when removing [4] or [5] where statistical significance is reached -0.27 [-0.50; -0.01] (Odds ratio 0.76) in favour of duloxetine.

Analyses made on the subgroup of fluoxetine studies (where the number of analysed patients was greater than 20), gave for the efficacy 0.09 [-0.09;0.26] (13 fluoxetine studies) still favouring fluoxetine, for the response factor -0.22 [-0.46;0.02] (10 fluoxetine studies) still favouring duloxetine and for the dropouts factor -0.02 [-0.33;0.28] (7 fluoxetine studies) similar results were found.

*For duloxetine compared with venlafaxine*, *cf*. [Table 4] investigating the efficacy score (in the effect size scale) the effect size varied from 0.16 to 0.25 favouring venlafaxine significantly in all analyses except when adjusting for sex repartition where the result is borderline significant 0.16 [-0.01;0.33] though still numerically favouring venlafaxine. The residual between-study variance is small in all analyses, the model which has the best fit (smallest residual between-study variance) gave an estimated effect size of 0.25 [0.11;0.40] significantly in favour of venlafaxine. Investigating the response factor, the odds ratio varied from 1.75 to 2.46 favouring venlafaxine significantly in all analyses. The residual between-study variance remained stable, the best fit (smallest residual between-study variance) corresponds to an odds ratio of 1.75. Concerning the dropouts the odds ratio varied from 1.14 to 1.30 throughout adjustments favouring numerically venlafaxine in all analyses. The residual between-study variance remained stable and small.

When removing studies one at a time from the analysis set, the conclusions didn't change thus favouring robustness in results.

## Discussion

The use of the meta-regression method to indirectly compare duloxetine with each active comparator revealed that there was no significant difference with fluoxetine either in efficacy or in safety. Findings only suggest that more patients might respond to duloxetine. Results suggest that duloxetine might be significantly less effective compared with venlafaxine, (in terms of treatment effects and number of response) with similar dropouts rates.

Results given by sensitivity analyses showed relatively good consistency, as no analysis changed the conclusions. The results became nonsignificant in one analysis comparing venlafaxine with duloxetine, but the estimated value seldom moved. When removing [4] or [5] from the analysis set, duloxetine treated patients had statistically more chance to respond than when treated with fluoxetine. These findings were obtained by removing the less favourable studies for duloxetine, and we found no differences in the design or patients' characteristics that may explain why. These tests showing significance (when comparing fluoxetine to duloxetine) or non-significance (when comparing venlafaxine to duloxetine), as in every study where multiple testing is performed, may be due to a drop in statistical power, which can bias the conclusions. As some robust trends have been found between the different drugs, the findings are considered robust to the confounding factors that have been investigated.

Our findings should, however, be interpreted with caution. Findings of superior efficacy by indirect comparisons are observational and therefore vulnerable to bias. Yet, several articles have recently shown that indirect comparisons adjusted at the aggregate level usually agree with direct comparisons. An indirect meta-analysis of studies comparing olanzapine with haloperidol and risperidone with haloperidol yielded conclusions similar to those found in a direct comparative randomized clinical trial of olanzapine and risperidone [43]. Song *et al*. [44] demonstrated that the results of adjusted indirect comparisons were usually similar to those of direct comparisons. In their study, there were a few significant discrepancies between the direct and the indirect estimates, although the direction of discrepancy was unpredictable. The authors concluded that empirical evidence presented in their study clearly indicates that in most cases, results of adjusted indirect comparisons are not significantly different from those of direct comparisons.

While we recognize that none of the trials involving duloxetine used venlafaxine as an active comparator, our results are in accordance with a recent meta-analysis comparing duloxetine and venlafaxine in the treatment of MDD [45] and a review comparing second-generation antidepressants [46].

Vis *et al*. used results of 6 trials with duloxetine and 4 with venlafaxine to report the efficacy and safety of either venlafaxine or duloxetine compared with placebo. They found that venlafaxine rates for remission and response were respectively 17.8% (CI_95% _9.0–26.5) and 24.4% (CI_95% _15.0–37.7) greater than placebo, compared with 14.2% (CI_95% _8.9–26.5) and 18.6% (CI_95% _13.0–24.2) for duloxetine. Reported adverse events were comparable between active drugs. The authors concluded that venlafaxine showed a favorable trend in remission and response rates compared with duloxetine, but that no significant between-drug differences were observed for dropout rates and adverse events. Due to the nature of the methodology used, no objective evidence concerning how venlafaxine performs when compared with duloxetine can be drawn. Nonetheless, the numerical trend seen in this paper is in accordance with the ones found here.

A review of second-generation antidepressants' efficacy in the treatment of MDD by Hansen *et al*. [46] found that significantly more patients responded to venlafaxine than to fluoxetine. The relative benefit: 1.12 (CI_95% _1.02–1.23) favoured venlafaxine. This result suggest the same pattern found here; response rates of venlafaxine are superior to duloxetine which are equal to fluoxetine

Concerning available comparisons with fluoxetine, of the 9 randomized clinical trials that evaluated the efficacy and safety of duloxetine, only two used fluoxetine as an active comparator [4,9]. Neither of these studies was specifically designed and powered to facilitate head-to-head comparisons between duloxetine and fluoxetine. The primary goal was comparison of duloxetine *vs*. placebo. These two studies (powered 65%) were identical parallel group, double-blind, forced-titration active- and placebo-controlled studies comparing duloxetine titrated from 20 mg to 60 mg BID with placebo over 8 weeks of acute treatment. A fluoxetine 20 mg QD arm was used as an internal active comparator standard. In these studies, duloxetine was statistically significantly superior to placebo on the primary analysis (mean change analysis from baseline of the HAMD-17 total score) and for some of the secondary endpoints. There was no statistically significant difference between fluoxetine and placebo for mean change in HAMD-17 total score in any of the studies. The fluoxetine treatments groups were underpowered qualitative control arms: [1] half patients included compared with duloxetine and placebo reaching low numbers (33 [9] and 37 [4]), [2] comparison of a fixed dose at the minimum recommended range for fluoxetine (20 mg/day) with the highest tested dose for duloxetine (120 mg/day). Higher doses of fluoxetine may have proven more effective and a more robust comparison of duloxetine, and fluoxetine should include a broader and more optimal dose range for comparison. Furthermore, as fluoxetine has proven to have an effect when compared with placebo [47,48], these direct comparisons are not sufficient to draw conclusions about duloxetine's superiority over fluoxetine.

Superiority of one antidepressant medication relative to another needs to be established by means of prospectively designed, adequately powered, head-to-head clinical trials. As the results of placebo-controlled trials are often sufficient to acquire the regulatory approval of new drugs, pharmaceutical companies may not be motivated to support trials that compare new drugs with existing active treatments. Lack of evidence from direct comparison between active interventions makes it difficult for clinicians to choose the most effective treatment for patients [49]. Because of the lack of direct evidence, indirect comparisons have been recommended [50]. Adjusted indirect comparison is a way to compare two compounds through their relative effect *vs*. a common comparator (placebo in our study). The indirect approach to meta-analysis requires certain conditions to yield optimal results. Differences in study designs, inclusion/exclusion criteria, patients characteristics at baseline as well as difference in drug dosage [48] and publication bias are limitations that may lead to unbalanced conclusions [43] and merit discussion.

Our study had some limitations. First, the time frame differs between active drugs. Because fluoxetine is the oldest antidepressant compared with venlafaxine and duloxetine, inclusion criteria for MDD was based on DSM III or IIIr criteria (not DSM IV) in the majority of the fluoxetine studies compared with those of venlafaxine and duloxetine. Secondly, sample sizes seem to be smaller for the fluoxetine studies and include patients with lower HAM-D score (14 to 19). Thirdly the patients characteristics, even if they vary only slightly can act as confounding factors and bias the results. Fourthly, dosages varied between studies and between drugs. Lastly, the missing data might not be balanced between treatments. All these sources of heterogeneity could lead to bias. Considering that the computation of an effect size included adjustment for baseline severity differences and that influence of patient characteristics and study designs were assessed through sensitivity analyses, some confidence can be put on the results if they show stability over the different analyses. Also, the random effect nature of the model used here should be able to deal with the remaining amount of bias that couldn't be measured or properly modelled. Finally, the other major issue in any meta-analysis is the potential publication bias. Publication bias is a major source of systematic bias in overviews, where trials with positive results are more likely to be published than those with neutral or negative results, especially if the trials are small. We therefore tested for publication bias using the Egger test for funnel plot asymmetry [51]. Ruling out completely publication bias is nearly impossible. Even so, any bias would most likely be in favour of the newer drug and its existence would not undermine the results presented here [52].

## Conclusion

In the absence of a well-powered randomised placebo controlled direct comparison trial, meta-regression analysis offers the most rigorous evidence science can buy. Even if it's true that the level of evidence provided by indirect comparisons is lower than the level provided by direct comparisons; in some cases [43] indirect comparisons have actually been able to predict the results of head-to head-clinical trials. The capacity of prediction is nonetheless directly linked to the quality of the methodology used and the information available. Both have been discussed in the core of this paper, and in this context the results seem stable enough to be confident that the bias are controlled and that the results provide valuable additional information to health care professionals, health economists and the pharmaceutical industry. These results suggest evidence of venlafaxine superiority compared with duloxetine and absence of a difference between fluoxetine and duloxetine. In any case, investigating the relative efficacy of duloxetine compared directly with other existing antidepressants – particularly venlafaxine – in a well-designed trial would be welcomed to challenge or reinforce our findings.

## Authors' contributions

Each author has made substantial contributions at every phase in the planning and writing of the manuscript. Each have each equally contributed to the drafting and critical revision of this work.

## Pre-publication history

The pre-publication history for this paper can be accessed here:


